# Colonic perforation during barium enema in a patient without known colonic disease: a case report

**DOI:** 10.4076/1757-1626-2-6716

**Published:** 2009-08-14

**Authors:** Necdet Fatih Yaşar, Enver İhtiyar

**Affiliations:** Department of General Surgery, Eskisehir Osmangazi University Faculty of MedicineMeselik Campus, 26480 EskisehirTurkey

## Abstract

The barium enema is a safe and accurate diagnostic study of the colon but, in rare cases, causes complications, such as colonic perforation. A colon weakened by iatrogenic trauma due to the enema tip and/or retention balloon, or by disease is more likely to perforate during an enema than is a normal healthy bowel. Rarely the colon may burst due to excessive transmural pressure alone. We report a case of colonic perforation during barium enema in a 72-year-old female patient, due to excessive barium applied into the rectum.

## Introduction

The barium enema is a safe and accurate diagnostic study of the colon but, in rare cases, complications, such as colonic perforation, barium impaction, water intoxication, allergic reactions, and cardiac arrhythmias, may result. Perforation of the bowel is the most frequent serious complication, occurring in approximately 0.02% to 0.04% of patients [1]. Intraperitoneal perforation is especially devastating, due to the combination by barium and bacterially loaded faecal material which causes severe, acute peritonitis with intravascular volume depletion. Adequet resuscitation, prompt fluid replacement and laparotomy for early resection or primary repair with an aggressive effort to evacuate barium as much as possible are essential. If the patient survives the initial shock and sepsis, later complications caused by dense intraperitoneal adhesions may develop.

Rarely the colon may burst due to excessive transmural pressure alone. However, a colon weakened by iatrogenic trauma or disease is more likely to perforate during an enema than is a normal healthy bowel. Injury to the rectal mucosa or anal canal due to the enema tip or retention balloon is probably the most common traumatic cause of barium enema perforation [1]. We report a case of barium enema perforation due to excessive pressure of barium.

## Case presentation

A 72-year-old, Turkish, Caucasian female patient was referred immediately to our surgery department after extravasation was found during barium enema procedure. The patient presented with complain of nausea, vomiting and intermittant cramping abdominal pain. Her vital signs included temperature of 37.4°C, blood pressure of 110/70 mmHg, pulse rate of 82 beats/min, respiratory rate of 24 breaths/min. On physical examination, the abdomen was distended and tenderness was noted to direct and rebound palpation with guarding in all quadrants. Routine hematological and biochemical investigations were within normal limits except for raised total leucocyte count (12,800/mm³). Plain X-ray of abdomen revealed dilation of rectum, sigmoid and descending colon with barium which occupied the abdomen intraperitoneally. A chest X-ray did not reveal any pneumoperitoneum.

Immediate fluid resuscitation and intravenous antibiotics were initiated. The patient underwent surgery after the diagnosis of acute abdomen was made. Exploratory laparotomy revealed perforation of the colon with a diameter of 10 cm, located 15 cm above the peritoneal reflection and barium covering the colon segments and the omentum. After an effective abdominal washout, sigmoid colon resection with Mikulicz colostomy was performed, followed by omentectomy because barium on the omentum could not have been removed by washout. Oral diet was given 24 hours after removing the nasogastric tube on the third postoperative day. On the ninth day of her hospitalization, the patient was discharged with complete recovery with a plan to close the colostomy 8 weeks later.

## Discussion

Tadros and Watters suggested four mechanisms of injury: trauma from the enema, overinflation of the balloon, recent colonoscopic instrumentation especially associated with biopsy and the presence of rectal mucosal disease such as cancer, stricture, diverticulosis or inflammatory bowel diseas [2]. Rarely the colon may burst due to excessive transmural pressure alone [1]. Our patient did not have any known colonic disease nor any recent colonoscopic instrumentation. Overinflation of the balloon was not recorded during the barium-enema examination by her doctor and trauma from the enema on the mucosa was not suspected because the perfortion site was 15 cm above the peritoneal reflection. Impairment of the tensile strength of the bowel wall due to her age could be the only risk factor for her. However, in our patient, the plain X-ray of abdomen revealed excessive amount of barium which dilated the rectum, sigmoid and descending colon accompanied by extravasation. The probable cause of the excessive amount of the intraluminal barium and colonic perforation was the generation of excessive pressure during the procedure. Besides the potential risk factors, such as injury to the rectal mucosa or anal canal due to the enema tip or retention balloon, excessive transmural pressure may be the only reason of the perforation during barium enema as in our case. Fry et al and Williams and Harned suggest that the incidence of colorectal perforation during barium-enema radiography can be reduced by 1) performing proctoscopy prior to barium enema, 2) avoiding the use of the rectal balloon in patients with known rectal lesions, using a safe tip-balloon design and inserting it after a careful digital rectal examination 3) avoiding barium studies in patients with active colitis, 4) delaying the examination by at least six days in cases of deep biopsy or polypectomy, 5) avoiding generation of pressure greater than that created by a column of barium suspension of one meter, and 6) using a lower concentration of barium when possible [1,3].

Patients with rectal perforations manifested by air extravasation can be successfully treated with intravenous antibiotics and complete bowel rest whereas patients with barium extravasation are treated with immediate operation and colostomy [3]. In our case, sigmoid colon where the peforation site was explored, was resected and Mikulicz colostomy was constructed and anastamosis was delayed for 8 weeks. Extraperitoneal perforation is usually less catastrophic but may result in pain, sepsis, cellulitis, abscess, rectal stricture, or fistula. Intramural extravasation often forms a persistent submucosal barium granuloma which may ulcerate or be mistaken for a neoplasm. The most dramatic complication of barium enema is venous intravasation of barium. Fortunately, this is quite rare as it may be immediately lethal [1].

As a conclusion, the colon may be perforated due to excessive transmural pressure alone and caution should be exercised to avoid any risk of perforation and excessive pressure should be avoided during the procedure.

**Figure 1.**
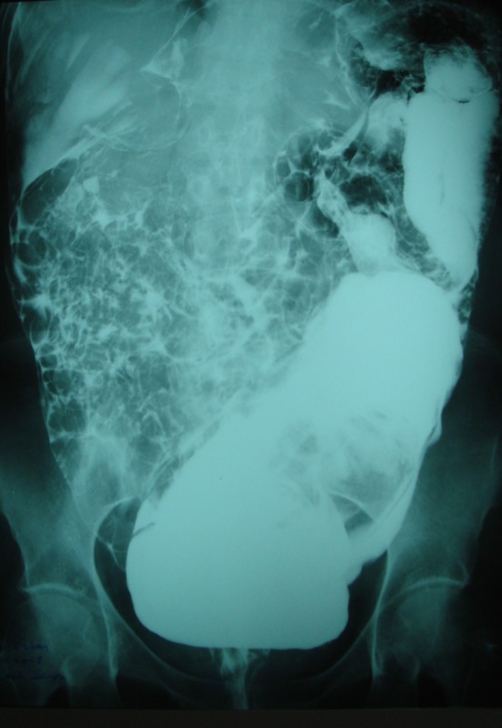
Anterior view of the abdomen in a plain radiograph showing a large amount of barium distending the rectum, sigmoid and descending colon and dispersing intraperitoneally during the enema procedure.

**Figure 2.**
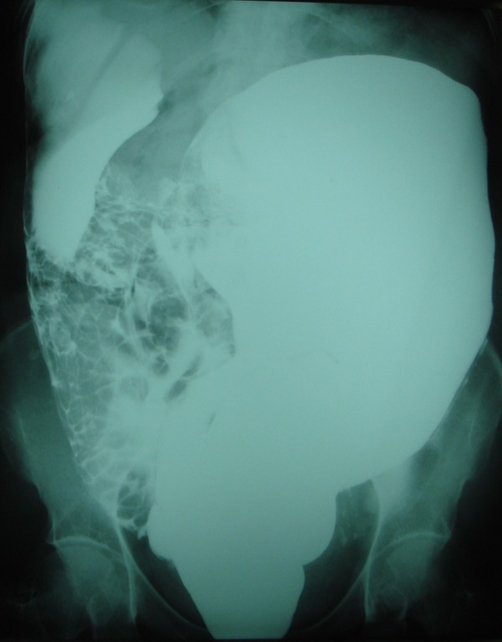
Lateral view of the abdomen in a plain radiograph showing a large amount of barium distending the rectum, sigmoid and descending colon and dispersing intraperitoneally during the enema procedure.

**Figure 3.**
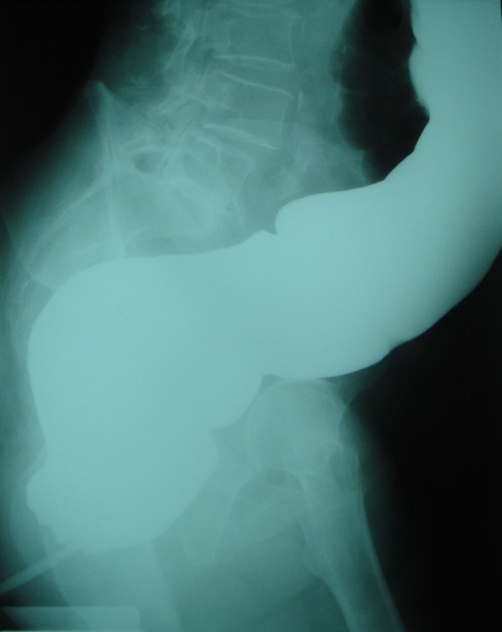
Lateral X-ray of the abdomen revealing barium in the distended colon.
